# Research on the mechanical behaviour of shale based on multiscale analysis

**DOI:** 10.1098/rsos.181039

**Published:** 2018-10-24

**Authors:** Qiang Han, Zhan Qu, Zhengyin Ye

**Affiliations:** 1 College of Petroleum Engineering/Postdoctoral Innovation Base, Xi'an Shiyou University, Xi'an 710065, People's Republic of China; 2 Shaanxi Key Laboratory of Well Stability and Fluid & Rock Mechanics in Oil and Gas Reservoirs, Xi'an Shiyou University, Xi'an 710065, People's Republic of China; 3 School of Aeronautics/Postdoctoral Research Station, Northwestern Polytechnical University, Xi'an 710072, People's Republic of China

**Keywords:** shale, multiscale analysis, mechanical evaluation, characterization method

## Abstract

In view of the difficulty in obtaining the mechanical properties of shale, the multiscale analysis of shale was performed on a shale outcrop from the Silurian Longmaxi Formation in the Changning area, Sichuan Basin, China. The nano-/micro-indentation test is an effective method for multiscale mechanical analysis. In this paper, effective criteria for the shale indentation test were evaluated. The elastic modulus was evaluated at a multiscale and the engineering validation of drilling cuttings was performed. The porosity tests showed that the pore distribution of shale from the nanoscale to macro-pore could be better displayed by the nuclear magnetic resonance test. The micro-scale elastic modulus and hardness increased nonlinearly with the increase in the clay packing density. It was observed that the size effect of the micro-hardness was based on porosity and composition. The partial spalling of shale at the micro-scale could lead to irregular bulges or steps in a load–displacement curve. The elastic modulus of pure clay minerals was 24.2 GPa on the parallel bedding plane and 15.8 GPa on the vertical bedding plane. The contact hardness (pure clay minerals) was 0.51 GPa. The indentation results showed that the micro-elastic modulus of shale obeyed the normal distribution, and the statistical average could predict the macro-mechanical properties effectively. The present work can provide a new way to recognize the mechanical behaviour of shale.

## 1. Introduction

Shale is a multicomponent rock that contains nanoscale porosity, clay particles, non-clay inclusions and perhaps even a small amount of kerogen. The composition diversity of shale plays an important role in a diverse set of mechanical properties [1–3]. The research on shale mechanics has mainly been conducted as follows. Parameters such as pore pressure and lithology can be used for seismic exploration and tectonic interpretation. During the drilling process, mechanical research of shale can be applied to the wellbore stability analysis as well as the optimization of the drilling bit and the drilling parameter. Underground safety construction can be effectively guaranteed, and drilling costs can be saved based on the study of formation of rock mechanics. For the hydraulic fracture process, the strength and fracture analysis of shale may drive a more scientific method for hydraulic fracture design and proppant optimization of shale gas reservoirs [4,5]. The main challenge is to identify a way to effectively obtain the mechanical properties of shale in the early stages of the exploration, drilling and development processes. This is essential to ensure the safe and efficient development of shale oil and gas. Currently, conventional mechanics experiments and appropriate failure criteria are generally adopted for shale mechanical evaluation [6]. However, there are some problems in conventional mechanicals experiments such as the large sample size, long time consumption and low interpretation accuracy. In addition, it is hard to evaluate the micromechanical behaviours of shale [7,8]. The multicomponent and microstructure of shale poses a challenge to log interpretation. Instability of a shale borehole increases the risk of logging instruments becoming stuck and the cost of effective drilling.

In the recent years, with the development of surface evaluation technology, the multiscale analysis has been the research focus at home and abroad. It is an interdisciplinary technique used in many fields such as physics and mechanics [9]. The research scale is usually divided into nano, micro and macro. An important significance of this study was to explain the intrinsic relationship between the microstructure and macro-behaviours of the material. Another aspect was to evaluate the multiscale mechanical properties of the material. The purpose was to predict the macro-behaviours of the material or to improve the material properties through microstructure design [10]. The study on macro-scale is based on the continuous medium mechanical test and inductive method. Meso/micro-scale mainly discusses the local stress/strain field. The actual structure and stress field of material are predicted by local homogenization field. There are two aspects to multiscale mechanical research. One is the material stiffness tensor where the elastic modulus and plastic characteristics of composite materials are studied. The other is the material failure and failure mechanism. The structural deterioration and the destruction mechanism are revealed by the minor damage analysis of the material. At the beginning of this century, the multiscale method was used to evaluate shale mechanics. The elasticity characteristic of shale at the nanoscale was evaluated by the homogenization model of the local tensor [11]. The microporous clay strength of shale was determined by dimensional analysis [12]. The strength model for cohesive frictional material, obtained by a variational method, provided a way to analyse the relationship between hardness and component of the nanoscale [13].

As one of the important means of multiscale mechanics research, the nano-/micro-indentation test provides a new way to evaluate the mechanical properties of shale. The test loads range from nanonewtons to newtons, and the indentation displacement range from nanometres to micrometres. The mechanical parameters, such as elastic modulus, hardness and yield strain, can be obtained by indentation analysis [14]. A detailed understanding of the indentation mechanical behaviours of the shale nanoparticle was provided by applying a statistical mechanics method [15]. Based on the theoretical analysis, the elastic properties of shale at the nanoscale were evaluated and the effective elastic tensor of the drainage porous clay was defined [16–19]. Mechanical properties of shale, such as elastic modulus, hardness and strength, were evaluated based on the micro-indentation test [20]. Subsequently, the influencing factors of the shale micro-indentation test were evaluated [21]. As a comprehensive parameter, indentation hardness was the overall performance of the local mechanical response under certain conditions. Indentation hardness can be related to strength and fracture by dimensional analysis and the finite element. The indentation test showed that hardness increased (or decreased) with the increase of the load [22,23]. This phenomenon is called the hardness size effect. The indeterminate of hardness affects the evaluation of shale multiscale strength. The measurement of evaluating the hardness size effect mainly includes the Meyer model, the Hays–Kendall model, the proportional specimen resistance (PSR) model and the PSR-modified model [24–26]. The PSR model and its modified model can be used for the analysis of brittle materials. Little has been reported on the hardness size effect of shale micro-indentation.

Additionally, the nano-/micro-indentation test is affected by many factors due to the high precision of the instrument. It can be summarized as follows: (i) measurement instruments including the indenter tip passivation, contact zero point and flexibility; (ii) sample properties including the indentation deformation mode, the degree of surface roughness and hygroscopicity; and (iii) indentation load, the maximum vertical load required for the experiment. According to the micro-indentation measurement, the influencing factor analysis of shale, such as effective load, surface hardening and post-elastic effect, was performed [20].

It can be seen that the indentation experiment and theoretical modelling of the multiscale mechanics have been preliminarily studied; however, there are still some problems that have to be solved. First, the mechanical parameters of shale are difficult to obtain in the drilling horizontal segment. Next, there is no systematic way to evaluate the effective measurement of the shale nano-/micro-indentation test. Next, the multiscale relationship between the composition and mechanics of shale is not clear. Finally, the engineering mechanics measurement of the drilling cuttings is not clear. In this work, the nano-/micro-indentation test of shale from the Longmaxi Formation was performed and analysed. The macroscopic mechanical properties of shale were predicted based on the micro-indentation test. The research provided a framework for recognizing the relationship between the composition and mechanics. The timely recognition of the mechanical state of the shale reservoir in the downhole is of great practical significance. Conventional mechanics experiment such as uniaxial/triaxial test can be replaced by indentation test gradually. The mechanical parameters of underground rock can be evaluated by the indentation test of the drill cuttings. It is of guiding significance for drilling optimization and wellbore stability.

## 2. Material and test

### 2.1. Multiscale composition of shale

As a diverse class of sedimentary rock, the mechanical properties of shale are related to mineral composition and pore structure. Below the composition scale, shale can be divided into four levels (table 1). The clay mineral is a basic unit composed of clay particles, with thickness to the order of 20 nm and the size of the order of 10^−9^ m. Nanoscale refers to a scale of the order of 10^−8^ to 10^−6^ m. This scale focuses on clay minerals and inter-granular pores, and the size ranges from 10 nm to 2 µm. The micro-scale refers to a scale of the order of 10^−5^ to 10^−4^ m and focuses on clay minerals and inclusions [15]. The macro-scale refers to a scale of the order of 10^−3^ and above.

**Table 1.** Multiscale component classification of shale.

classification	scale (m)	typical characteristic
clay mineral	≤10^−9^	approximately equal particle and the thicknesses of the order of 20 nm
nanoscale	10^−8^ ∼ 10^−6^	porous clay, curved or stitched intergranular pores
micro-scale	10^−5^ ∼ 10^−4^	clay mineral and inclusion
macro-scale	≥10^−3^	macroscopic sample

According to the multiscale composition classification, shale is composed of three parts: clay minerals, non-clay minerals and pores. The volume fraction of the composition is calculated as

2.1 $$f_{\mathrm{inc}}+f_{c}+\varphi =1,$$

where *f_c_* is the volume fraction of clay mineral; *f*_inc_ is the volume fraction of non-clay mineral; and *φ* is porosity. For shale at the nanoscale, the volume fraction of clay is written as

2.2 $$f_{c}=(1-\varphi )\frac{\sum_{i=1}^{\mathrm{CM}}\left(m_{i}/\rho_{i}\right)}{\sum_{i=1}^{N}\left(m_{i}/\rho_{i}\right)},$$

where CM is the clay mineral phase; *N* is the shale mineral phase of each constituent; and *m_i_* is the mass fraction of a constituent with density *ρ_i_*. The volume fraction *f*_inc_ is given by

2.3 $$f_{inc}=(1-\varphi )\frac{\sum_{i=1}^{\mathrm{NC}}\left(m_{i}/\rho_{i}\right)}{\sum_{i=1}^{N}\left(m_{i}/\rho_{i}\right)},$$

where NC is the non-clay phase such as quartz, feldspar, plagioclase, calcite, dolomite and pyrite.

The clay packing density can be expressed in terms of the composition fraction and porosity by

2.4 $$\eta =\frac{(1-\varphi )\sum_{i=1}^{\mathrm{CM}}\left(m_{i}/\rho_{i}\right)}{\sum_{i=1}^{\mathrm{CM}}\left(m_{i}/\rho_{i}\right)+\varphi \sum_{i=1}^{\mathrm{NC}}\left(m_{i}/\rho_{i}\right)}=\frac{\;f_{c}}{1-f_{\mathrm{inc}}}.$$

**Figure 1.**
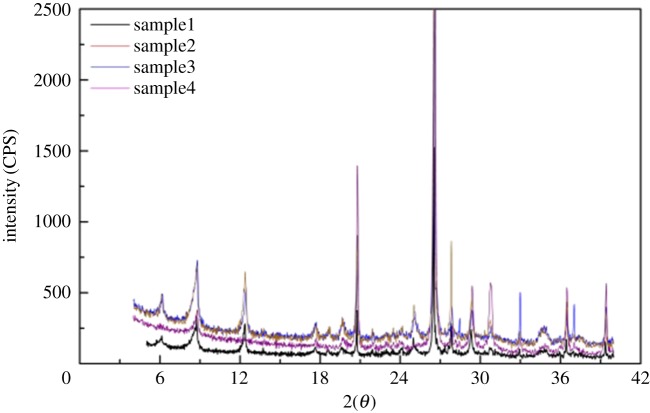
Typical spectrum of shale XRD diffraction.

In this paper, the shale samples were from the Longmaxi Formation shale outcrop and drilling cuttings in the Sichuan Basin. The organic carbon content of the Longmaxi Formation is mostly from 1% to 2%, and is 1.53% on average [27]. The vitrinite reflectance of the Longmaxi Formation ranges from 2% to 3.3% [28]. X-ray diffraction (XRD) was used to study the structure of shale (figure 1) and the results showed that Longmaxi shale is mainly composed of clay, quartz, calcite, dolomite, pyrite and a very small amount of potassium feldspar and plagioclase (table 2). It can be seen that the shale of the Longmaxi Formation is mainly composed of medium-hardness minerals.

**Table 2.** Total mineral composition of shale in the Longmaxi Formation.

	mass percentages of shale (%)						
sample no.	clay	quartz	orthoclase	plagioclase	calcite	dolomite	pyrite
1	33.3	17.5	2.3	4.1	33.8	8.2	0.8
2	33.1	14.7	1.1	1.7	24.9	2.7	21.8
3	54.2	32.2	2.2	7.0	3.6	0.8	/
4	39.3	31.6	/	3.1	7.0	10.2	8.8

**Figure 2.**
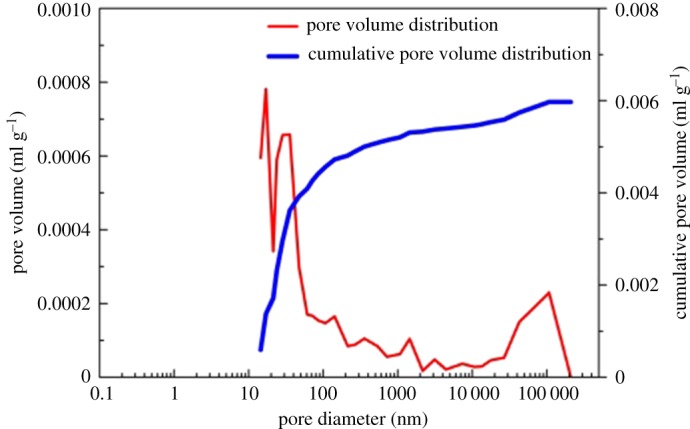
The pore size distribution of the shale results from the high-pressure mercury intrusion test.

**Figure 3.**
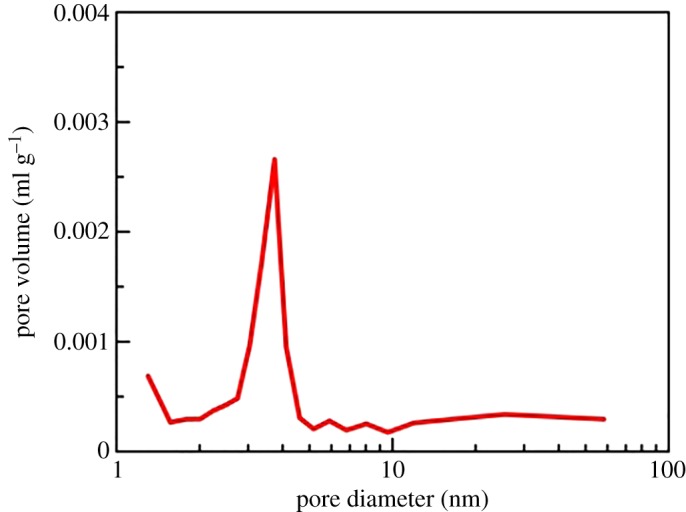
The pore size distribution of the shale results from the low-pressure nitrogen adsorption test.

**Figure 4.**
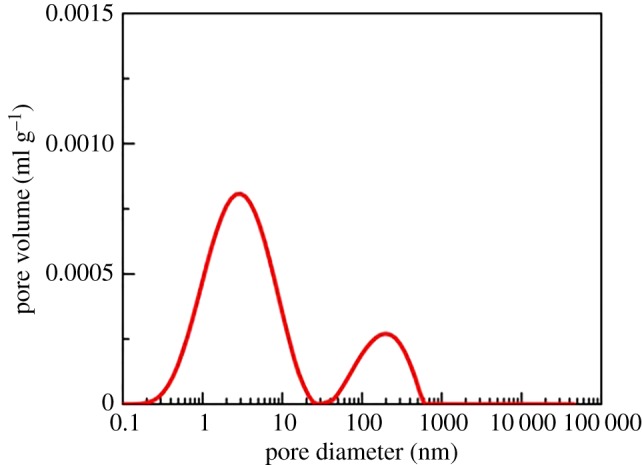
The pore size distribution of shale results from the NMR test.

To evaluate shale porosity, three types of experiments were performed. The results of high-pressure mercury intrusion showed that the pore size was mainly 10–1000 nm, which had an obvious effect on the macro-pores and micro-pores (figure 2). The results from the low-pressure nitrogen adsorption showed that the pore size was 1–10 nm (figure 3), which had an obvious effect on the micro-pores and nano-pores. The result of the nuclear magnetic resonance (NMR) showed that the pore size was 0.1–100 nm (figure 4). The pore size measured by high-pressure mercury intrusion was related to the maximum working pressure. The problem was that mercury was not readily accessible to the nano-pores in shale. The adsorption amount of nitrogen was related to the micro-pore size of shale. The NMR test could better display the pore distribution from the nanoscale to macro-pore. As a consequence, all of the porosity in the shale could be well characterized by NMR (table 3). Mass fraction obtained by XRD was converted into the volume fraction. To obtain the clay packing density, the volume fraction and porosity obtained by NMR were combined into equation (2.4).

**Table 3.** The porosity of shale results from the NMR test.

no.	diameter (mm^−1^)	height (mm^−1^)	volume (ml^−1^)	porosity (%)
1	25.32	25.34	12.753	4.8
2	25.3	25.51	12.818	6.2
3	25.3	24.7	12.411	3.3
4	25.28	25.05	12.567	5.7

### 2.2. Equipment

Indentation experiments were performed on two different test platforms. The primary test platform was a Triboindenter used for the nano-indentation test. This platform determined the load from 0 to 10 mN. The load resolution was less than 1 nN. The maximum indentation displacement could reach 20 µm. Another platform was the MFT-4000 multifunction material tester that was for the micro-indentation test. This platform determined the load from 0.5 to 300 N. The load resolution was less than 0.5 N. The maximum indentation displacement could reach 200 µm.

As a key component of the indentation instrument, the indenter tip is designed to have a variety of shapes. The initial contact stress of the spherical indenter is small enough for it to be suitable for measuring the contact damage of soft material. A cylinder indenter is suitable for the measurement of large contact stiffness materials. The friction resistance between the pyramidal indenter and material can be ignored because the pyramidal indenter has as small radius of curvature. The common pyramidal indenter contains the Vickers indenter and the Berkovich indenter. According to the instructions of ISO 14577 and BG/T 24458-2008, the Berkovich indenter was chosen in this study because of its good self-similarity.

### 2.3. Sample

The shale sample for the nano-/micro-indentation test was from the Longmaxi Formation outcrop in the Changning area, Sichuan Basin. They were cut along the bedding direction and the size was 60 × 25 × 15 mm based on ISO 14577. In order to reduce the influence of surface roughness, the surface of the specimen was repeatedly polished before the indentation test. The test surface of the sample was perpendicular to the indenter.

### 2.4. Test procedure

**Figure 5.**
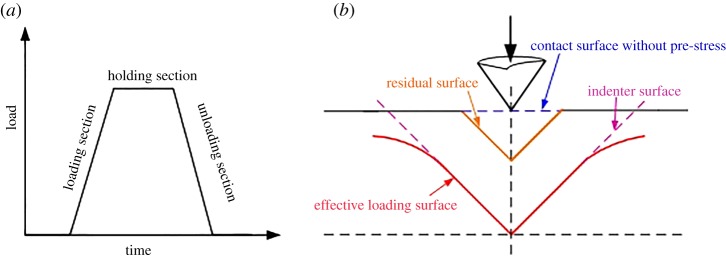
Typical indentation loading function and indentation change schematic diagram. (*a*) Indentation loading function; (*b*) typical indentation response.

**Figure 6.**
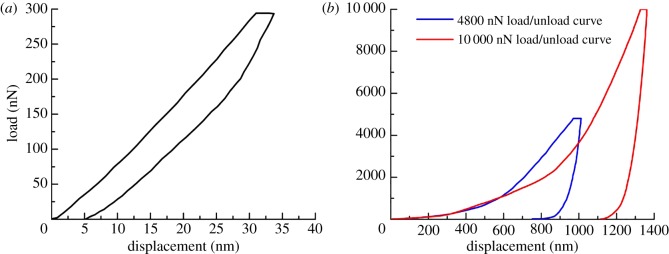
Typical nano-indentation load-displacement curve of shale. (*a*) 300 nN load-displacement curve; (*b*) 4800 nN and 10 000 nN load-displacement curve.

**Figure 7.**
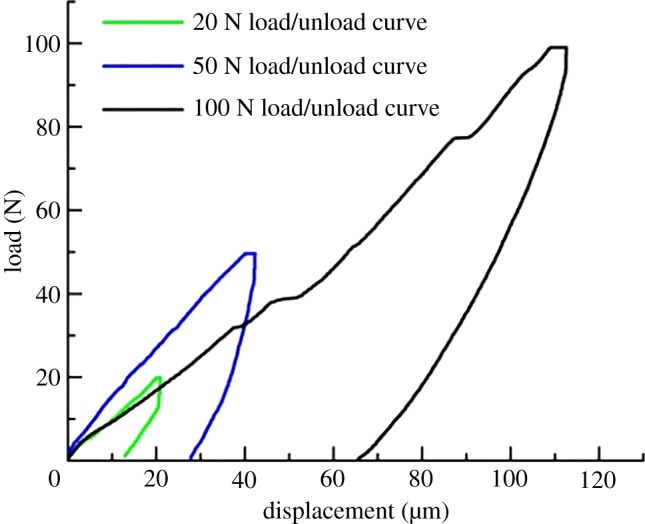
Typical micro-indentation load-displacement curve of shale.

The general procedure for an indentation test consists of four steps. First, the indenter comes into contact with the sample surface without pre-stress. Next, the indenter is pressed vertically into the sample surface with a constant load rate. When the maximum load is reached, the load is held for 15 s. Finally, the load is unloaded to zero at a constant rate and the indenter is lifted away from the sample surface (figure 5). Figures 6 and 7 display the load-displacement curves of the nanoscale and micro-scale, respectively.

Regarding the heterogeneity of shale, it is difficult to show the overall mechanical response of the sample based on a single indentation test. The grid indentation with statistical analysis is an approach that can evaluate the macroscopic mechanical response of shale. According to the Delesse criterion, the surface percentage of the sample is equivalent to the volume distribution percentage for a completely disordered material [29]. Therefore, the grid indentation test ensures that the mechanical behaviour of the sample is less relevant to the indentation position. Based on the standard and nano-/micro-indentation of shale, the distance between two nano-indentation points was 16 µm, and the distance between two micro-indentation points was 2 mm. In this way, the interference of the stress field near the indentation points was eliminated.

## 3. Results

As a high-precision experiment, the parameters of the nano-/micro-indentation test can be influenced by the tester, sample property and environment. The effective measurement parameters, abnormal curve and the mechanical evaluation method were studied in this section.

### 3.1. Nano-indentation mechanical response

**Figure 8.**
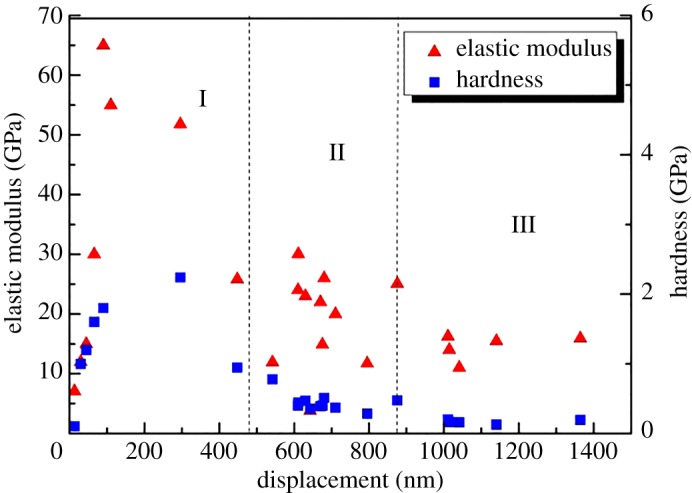
Relationship between elastic modulus, hardness and indentation displacement at a nanoscale.

The Oliver–Pharr method was used to obtain the elastic modulus and hardness based on the load-displacement curve analysis [30]. Figure 8 shows the mechanical response of the shale nano-indentation test. The change in the mechanical parameters (elastic modulus and hardness) with indentation displacement can consist of three regions. In region I, the elastic modulus and hardness of shale increase rapidly with the increase of indentation displacement and decrease rapidly after reaching a certain value. Region II shows that the elastic modulus and hardness are generally stable with the increase in indentation displacement. This stage plays an important part in the nano-indentation test. In region III, the elastic modulus and hardness decrease gradually with the increase in indentation displacement. In region III, the experimental results can be used for qualitative reference analysis as the indentation displacement is longer than the clay size.

The multiscale evaluation of shale was based on the principle of scale separation. The size of the clay mineral was assumed as *l*. The characteristic size of the representative elementary volume was assumed as *L*. The indentation displacement was assumed to be *h*. Continuum mechanics analysis showed that the separation of the scale condition must be achieved as *l* ≪ *L* ≪ 3*h*. In addition, *h* should be smaller than the length scale of shale to avoid the sample effect. The test should ensure that the displacement of most of the indentation is stable under the same load to satisfy the average response of the clay phase. The results of nano-indentation test showed that the load-displacement curve stayed smooth when the load reached 4800 nN, and the displacement was 700–900 nm. It could be seen that the mechanical properties of nano-indentation test were stable when the load was determined as 4800 nN, and can be used as a payload for the nano-indentation test of shale.

### 3.2. Micro-indentation mechanical response

**Figure 9.**
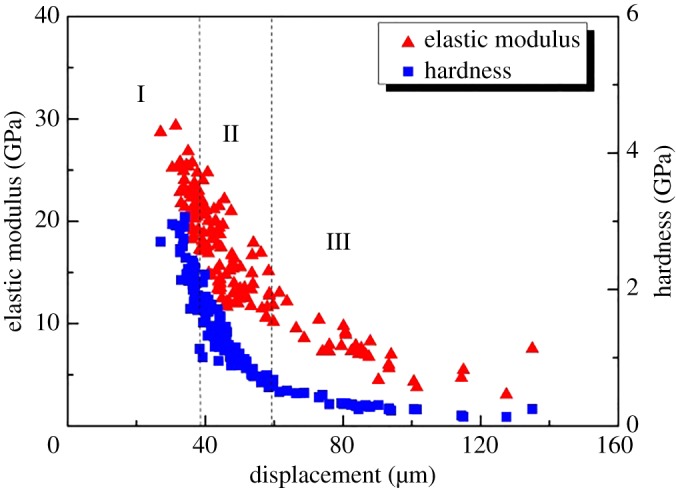
Relationship between elastic modulus, hardness and indentation displacement at a micro-scale.

The Oliver–Pharr method was also applied for the analysis of the micro-indentation test. Figure 9 displays the mechanical response for the elastic modulus and hardness. The elastic modulus and hardness exhibited a nonlinear decrease with the increase of indentation displacement. The change in the mechanical parameters (elastic modulus and hardness) with micro-indentation displacement can also consist of three regions [21]. In region I, the elastic modulus and hardness decrease rapidly with a small indentation displacement. In this stage, the curves of load-displacement were not similar. The main reason for this phenomenon was that the strain hardening was generated on the surface of the shale during the polishing process. Region II shows that the mechanical properties decrease gradually with the increase of indentation displacement. In region III, the variation of the mechanical properties is shown in the stage of indentation cracking. When the applied load reaches the rupture threshold, the crack is initiated in the peripheral region of the indentation. In this stage, the measured values decline steadily.

### 3.3. Hardness size effect of micro-indentation

**Figure 10.**
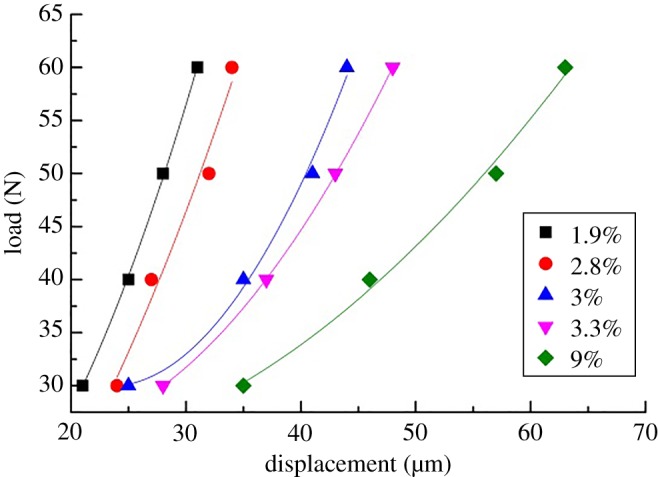
The load-displacement curve of various shale porosity (colours represent the porosity in the shale).

According to the results of the shale micro-indentation test, the hardness of the shale decreased gradually with the increase in the indentation displacement. This phenomenon is called the hardness size effect, and it plays an important role in the measurement. Hardness is a comprehensive characteristic parameter related to strength [22]. The uncertainty of indentation hardness can seriously affect the multiscale strength of shale. The main reason for this phenomenon is the composition and microstructure of shale. Figure 10 shows the relationship between the load and indentation displacement with a range of porosity in shale. It can be seen that the displacement was positively correlated with porosity under the same load.

The PSR and PSR-modified models were used to evaluate the shale hardness at a micro-scale [25,26,31]. According to the PSR model, the load of indentation, *P*, is calculated as

3.1 $$P(h)=a_{1}(6.749h)+a_{2}{\left(6.749h\right)}^{2},$$

where *a*_1_ is the coefficient of elasticity, *a*_2_ is the fitting parameter of the PSR model and *h* is the indentation depth. The first term on the right side of equation (3.1) is the surface energy of the material represented by displacement. The second term is the strain energy of the material represented by displacement. The counterforce is defined effectively to describe the physical meaning of the indentation. When the material shows micro-cracks caused by the load, part of the energy is consumed during the indentation test. In this case, equation (3.1) does not satisfy the equilibrium condition. In order to address this problem, the PSR-modified model is presented. The coefficient *b*_0_ is introduced to modify the influence of residual stress. The modified PSR model can be written as

3.2 $$P=b_{0}+b_{1}h+b_{2}h^{2},$$

where *b*_0_ is the coefficient of the residual stress on the surface; *b*_1_ is the coefficient related to the elastic properties of the materials; and *b*_2_ is the coefficient related to the load-depth hardness. According to the definition of hardness, the indentation response can be written as

3.3 $$H=\frac{P}{A},$$

where *H* is the hardness; and *A* is the contact area (the function of displacement and equivalent semi-conical angle of the indenter). The PSR model and the PSR-modified model were used to fit the curve in figure 10. Next, the fitting data obtained from the curve were used to determine the shale hardness with a variety of porosities. Tables 4 and 5 display a typical set of solutions for the PSR model and the PSR-modified model of the shale, respectively. The results showed that the hardness of the two models decreased with the increase in porosity. The correlation coefficient of the PSR-modified model was better than that of the PSR model. However, the average hardness from the indentation test did not match the solution of the PSR/modified model. This phenomenon showed that the hardness of shale was affected not only by the pores but also by the role of composition.

**Table 4.** PSR model fitting parameters of hardness.

*φ* (%)	*a*_1_	*a*_2_	correlation coefficient	H (GPa)
1.9	0.04657	0.00115	0.99978	2.1325
2.8	0.03269	9.713 × 10^−4^	0.96629	1.8011
3	0.12808	2.184 × 10^−4^	0.93196	0.4050
3.3	0.10975	2.2401 × 10^−4^	0.98539	0.4154
9	0.10609	7.7477 × 10^−5^	0.98193	0.1436

**Table 5.** PSR-modified model fitting parameters of hardness.

*φ* (%)	*b*_0_	*b*_1_	*b*_2_	correlation coefficient	H (GPa)
1.9	11.566	–0.08622	0.00152	0.99826	2.8187
2.8	0.58427	0.02664	9.867 × 10^−4^	0.93258	1.8297
3	67.2558	–0.47407	0.00151	0.98235	2.8001
3.3	34.65	–0.16901	7.6353 × 10^−4^	0.9992	1.4159
9	24.606	–0.04815	3.0541 × 10^−4^	0.98223	0.5663

### 3.4. Typical abnormal curve

**Figure 11.**
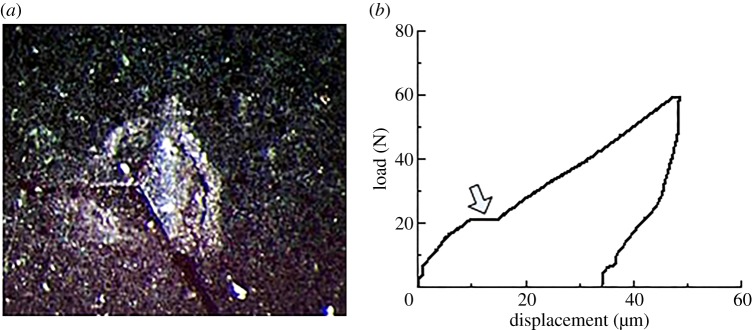
Characteristics of the micro-indentation point and load-displacement curve of 60 N. (*a*) Micro-indentation point and (*b*) load-displacement curve.

**Figure 12.**
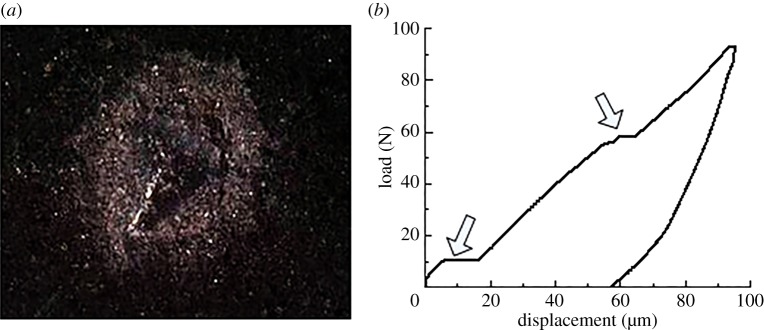
Characteristics of the micro-indentation point and load-displacement curve of 100 N. (*a*) Micro-indentation point and (*b*) load-displacement curve.

The indentation response is associated with the composition and microstructure of shale. When the plastic bearing limit is reached on the surface of the shale indentation, the high stress area shows stratification and buckling. As the load increases further, crack appears in the vicinity of the indentation along the radial or ring direction (figure 11*a*). Figure 11*b* displays the corresponding load-displacement curve. The response provides a horizontal step. As the load continues to increase, the material of the indentation area is peeled off. For repetitive loading on the spalling, the curve appeared as irregular bumps and steps (figure 12). The elastic modulus and hardness obtained from the abnormal indentation curve were not the true values. The abnormal curve should be avoided in the process of the indentation test.

### 3.5. Elastic modulus evaluation method of nano-/micro-indentation

The elastic modulus is part of the most commonly extracted parameters of the nano-/micro-indentation test. Three analytical methods for indentation analysis were introduced according to the standard BG/T 22458. The first was the Oliver–Pharr method, which is based on elastic contact theory. The next was the Cheng–Cheng method, which is based on dimensional analysis and the finite-element simulation [32]. The third was the Ma method, which is an energy analysis based on the Cheng–Cheng method [33]. As a general method, the mechanical mechanism of Oliver–Pharr method is clear. The test accuracy is affected by contact depth. The evaluation of Ma method is independent of contact depth, but is affected by contact stiffness. Cheng–Cheng method based on dimensional analysis requires a large number of experiments to correct the coefficient.

**Figure 13.**
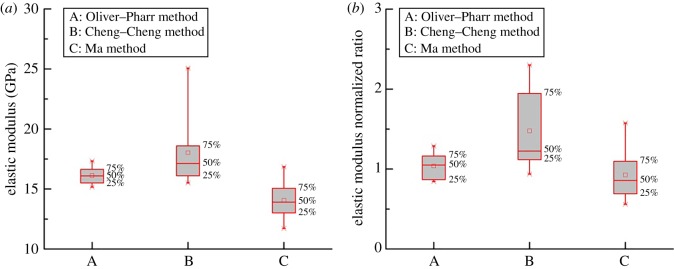
Elastic modulus comparison analysis of three methods. (*a*) Nanoscale and (*b*) micro-scale.

Figure 13*a* displays the elastic modulus of the nano-indentation obtained as a result of the three methods. The Oliver–Pharr method showed that elastic modulus was 15.16–17.33 GPa. The distribution deviation from 25% to 75% was less than 1.5 GPa. The Cheng–Cheng method showed that the elastic modulus was 15.51–25.07 GPa. The distribution deviation from 25% to 75% was less than 3.5 GPa. Based on the Ma method, the results showed that the elastic modulus was 11.72–16.85 GPa, and the distribution deviation from 25% to 75% was less than 3 GPa (table 6). In this study, the elastic modulus of the nano-indentation obtained by the Oliver–Pharr converged more than in the other methods. For shale micro-indentation, the reliability of the elastic modulus is determined by a comparison with the triaxial test. The mechanical triaxial test platform chose RTR-1000. The experimental result was agreed as a true value. Figure 13*b* and table 7 show that the normalization of the elastic modulus was 0.84–1.3 by the Oliver–Pharr method, 0.94–2.3 by the Cheng–Cheng method and 0.56–1.57 by the Ma method. In this study, the solution through the Oliver–Pharr method converged more than the other methods. The results showed that the Oliver–Pharr method was suitable for the shale nano-/micro-indentation test.

**Table 6.** Nano-modulus comparison of the three methods.

method	average value/GPa	standard deviation
Oliver–Pharr	16.12	0.74
Cheng–Cheng	18.02	3.09
Ma	14.06	1.61

**Table 7.** Normalized analysis of micro-modulus.

method	average value/GPa	standard deviation
Oliver–Pharr	1.04	0.17
Cheng–Cheng	1.46	0.51
Ma	0.89	0.34

## 4. Discussion

### 4.1. Clay minerals

Unlike many other minerals, the mechanical behaviours of clay minerals are evaluated based on the experimental experience fitting method. The main reason is that clay particles are too small to be tested in solid form. Experimental samples included crystal clay, and mixed clay and epoxy cemented clay (table 8) [34,35]. Since there is no consensus on testing clay minerals, the elastic behaviours of the clay minerals in the Longmaxi Formation are not clear.

**Table 8.** Properties of clay minerals [34,35].

clay minerals	sample types	experimental method	parameters
muscovite	crystal	/	*C*_11_ = 178, *C*_33_ = 55, *C*_12_ = 42, *C*_13_ = 15, *C*_44_ = 12
kaolinite	clay mixture	acoustic	*E* = 27.6, *v* = 0.3
dickite		AFAM	*E* = 6.2
kaolinite, smectite		acoustic (extrapolation)	*E* = 9.8–15.4, *v* = 0.25
illite	clay in epoxy resin	acoustic	*E* = 67, *v* = 0.32
smectite/illite			*E* = 47, *v* = 0.29
chlorite			*E* = 75–194, *v* = 0.3

### 4.2. Nanoscale of shale

To obtain the mechanical properties of the clay minerals, a nano-indentation experiment was carried out. The reduced modulus of the pure clay mineral obtained was 10–15 GPa based on the nano-indentation test [36]. The nano-indentation test of the Geogenome shale showed that the average of the elastic modulus was 21.4 GPa on the parallel plane, and the elastic modulus was 15.9 GPa on the vertical plane. The average of hardness was 0.56 GPa.

**Figure 14.**
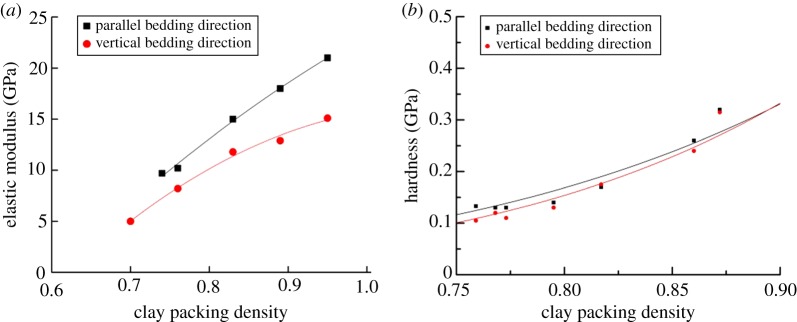
The relationship between shale nano-mechanics and clay packing. (*a*) Elastic modulus-clay packing curve and (*b*) hardness-clay packing curve.

Figure 14 displays the relationship between the average of mechanical parameters (*E*, *H*) and the clay packing density for the shale outcrops of the Longmaxi Formation. It was clear that the range of the elastic modulus was 5–20.3 GPa. The elastic modulus of the vertical plane was smaller than that of the parallel plane at the same packing density. It can be seen that the elastic modulus gradually increased with the increase of packing density. When the clay packing density reached 1 (without porosity), the intrinsic mechanical properties of clay were obtained by extrapolation. The results of the shale indentation test in the Longmaxi Formation showed that the average of the elastic modulus was 24.2 GPa on the parallel plane and was 15.8 GPa on the vertical plane. The average hardness was 0.51 GPa.

### 4.3. Micro-scale of shale

At the micro-scale, shale is clearly seen as a composite material consisting of non-clay inclusions and porous clay. The micro-mechanical behaviour of shale is significantly affected by the composition. As with the nano-indentation test, the elastic modulus of micro-indentation also increased with the increase of packing density. Moreover, in the average indentation elastic modulus, we found that the anisotropy of the multiscale was different. The anisotropy coefficient can be expressed as

4.1 $$t=\frac{E_{v}}{E_{h}}.$$

**Figure 15.**
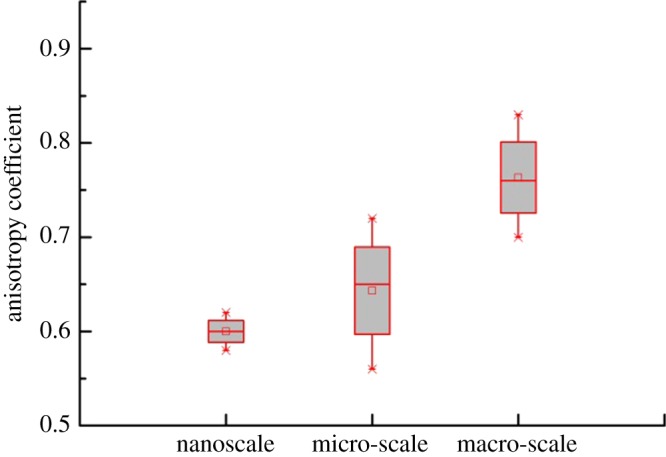
Elastic modulus anisotropy analysis.

Figure 15 shows the anisotropy coefficient of the multiscale (nano-micro-macro) obtained by the experiment. It was clear that the anisotropy coefficient was 0.58–0.62 at the nanoscale, 0.56–0.72 at the micro-scale and 0.7–0.83 at the macro-scale. It was observed that the elastic modulus of shale was anisotropic at all three scales.

### 4.4. Comparative analysis of macro-/micro-scale

**Figure 16.**
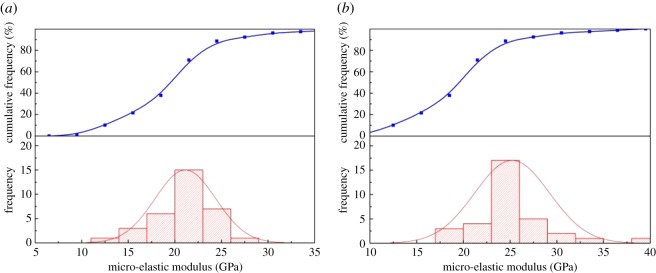
Probability statistics of micro-elastic modulus. (*a*) Vertical bedding direction and (*b*) parallel bedding direction.

**Figure 17.**
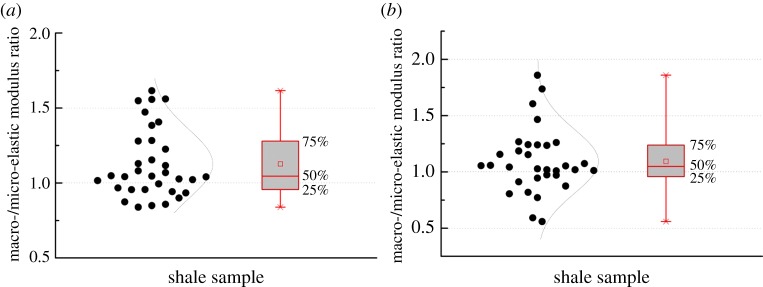
Distribution of macro-/micro-elastic modulus ratio of shale. (*a*) Vertical bedding direction and (*b*) parallel bedding direction.

The macroscopic test was performed using a RTR-1000 triaxial rock mechanical experiment tester. The test result was agreed as a true value to compare with the result of the micro-indentation test. The elastic modulus of shale was 22.7 GPa on the parallel plane, and 27.35 GPa on the vertical plane. Figure 16 shows the distribution of the shale elastic modulus from the micro-indentation test. The elastic modulus had a normal distribution, with the peak ranging from 20 to 23 GPa, and an average of 21.06 GPa on the vertical plane. The value of the peak was 22.5–26.5 GPa and had an average of 25.12 GPa on the parallel plane. Figure 17 displays the elastic modulus ratio for the macro-scale and micro-scale. The ratio of macroscopic to microscopic elastic modulus on the vertical bedding direction was 0.8–1.7, and 1.05 on average. The distribution deviation from 25% to 75% was less than 0.34. The ratio of macroscopic to microscopic elastic modulus on the parallel bedding direction was 0.52–1.8, and 1.1 on average. The distribution deviation from 25% to 75% was less than 0.3. The results demonstrated that the statistical average of the micro-elastic modulus could represent the macro-elastic modulus.

## 5. Engineering verification and application

**Figure 18.**
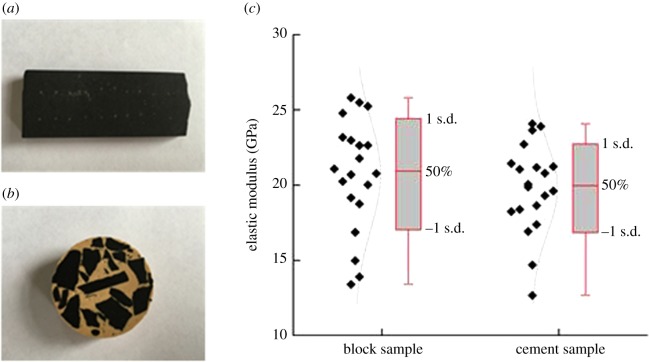
Analysis of indentation elastic modulus of block and cement sample. (*a*) Block sample, (*b*) Cement sample and (*c*) elastic modulus distribution of block sample and cement sample.

This study clarified the rationality to predict the shale macro-mechanical behaviours of the block sample by micro-indentation test. In this section, the indentation test of the drilling cuttings was evaluated. First, the micro-indentation test on the block sample of shale showed that the elastic modulus was 17–23 GPa, with an average of 21.4 GPa. Additionally, the block sample was broken (cutting's effective radius less than 0.5 cm) and cemented with epoxy resin. The test on the cemented sample showed that the elastic modulus was 16–22 GPa, with an average of 19.7 GPa (figure 18). It was clear that the two experimental results of the elastic modulus were similar, and the average of the block sample was slightly higher than that of the cemented one. The foremost reason was that the effects of micro-cracks on the block sample were produced during the destruction process. Therefore, it was feasible to evaluate the shale mechanical properties through cement cuttings.

**Figure 19.**
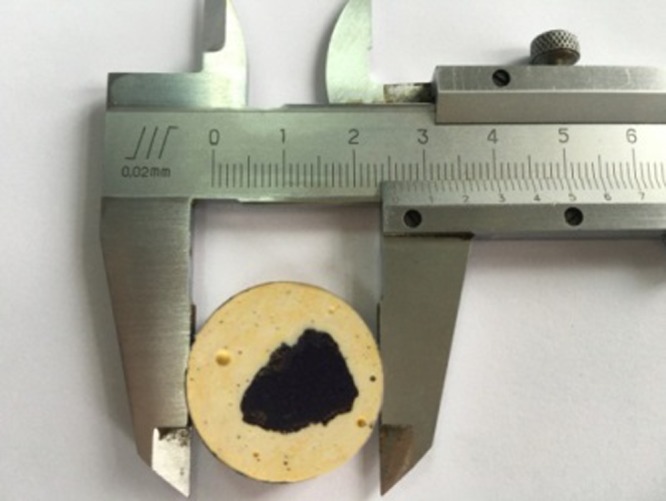
A sample of the cemented cuttings.

**Figure 20.**
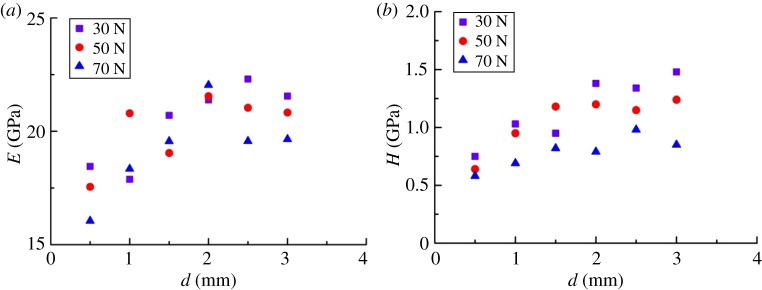
The distribution of mechanical parameters of the cuttings at the cementation boundary. (*a*) The boundary distribution of the elastic modulus of cuttings and (*b*) the boundary distribution of the hardness of cuttings.

**Figure 21.**
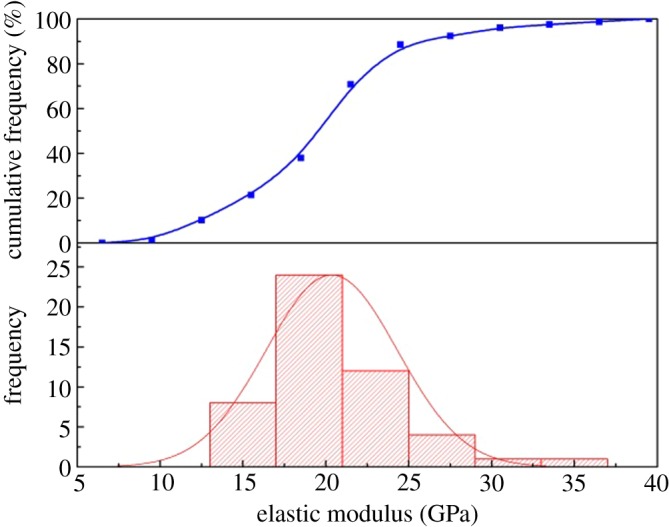
Probability statistics of the micro-elastic modulus on the vertical plane of the cuttings.

Drilling cuttings were obtained from 1500 to 1550 m underground at the Longmaxi Formation. First, the cuttings were washed and dried according to the standard of BGT-29172. Next, cuttings were cemented with epoxy resin. The radius of the cement sample was defined as 2.5 cm and the height was 2.0 cm (figure 19). Since the epoxy and shale cuttings were two dissimilar materials, the mechanical response near the contact area between the epoxy and shale cuttings must be analysed. Various loads (30 N/50 N/70 N) were selected for the micro-indentation test. Figure 20*a* displays the distribution relationship between the elastic modulus of the cuttings and cementation boundary. It was clear that the elastic modulus tended to fluctuate when *d* was less than 2 mm. Figure 20*b* displays the distribution relationship between the indentation hardness of the cuttings and cementation boundary. The influence of hardness was weaker than the effect on the modulus. The effect of the cementation transition zone (*d* < 2 mm) could not be ignored, where *d* is the distance between the indentation and the cementation boundary. The test showed that the elastic modulus of the drilling cuttings was 13.87–33.97 GPa, had a peak range of 15–20 GPa and an average of 20.2 GPa (figure 21). The well logging interpretation in the same horizon displayed that the average of the static elastic modulus was 21.35 GPa.

## 6. Conclusion

In this paper, nano-/micro-indentation tests of shale were performed, and the multiscale mechanical properties of shale were evaluated. The samples were taken from outcrops and underground drilling cuttings from the Longmaxi shale Formation in the Changning area in China. The results showed that the NMR test could better display the pore distribution from the nanoscale to the macro-pore. The nano-indentation tests showed that the elastic modulus and hardness of clay increased rapidly with the increase of indentation displacement and then decreased rapidly after reaching a certain value. The micro-indentation tests showed that the elastic modulus and hardness of shale gradually decreased with the increase of indentation displacement. The intrinsic mechanical properties of clay were obtained by extrapolation. The intrinsic elastic modulus of clay was 24.2 GPa on the parallel plane, and 15.8 GPa on the vertical plane. The average of hardness was 0.51 GPa. The elastic modulus and hardness of shale obtained by the micro-indentation test gradually decreased with the increase of indentation displacement. The macro-/micro-mechanical comparison showed that micro-indentation technology could be used to predict the macroscopic mechanical properties of shale. The elastic modulus of the drilling cuttings obtained by the indentation test correlated well with the log interpretation. It was observed that this technology could be applied to engineering mechanics prediction.

## Data accessibility

Data have been made publicly available in the Dryad Digital Repository: http://dx.doi.org/10.5061/dryad.rh094v7 [37].
